# Classification and Visualization of Chemotherapy-Induced Cognitive Impairment in Volumetric Convolutional Neural Networks

**DOI:** 10.3390/jpm11101025

**Published:** 2021-10-14

**Authors:** Kai-Yi Lin, Vincent Chin-Hung Chen, Yuan-Hsiung Tsai, Roger S. McIntyre, Jun-Cheng Weng

**Affiliations:** 1 Department of Medical Imaging and Radiological Sciences, Bachelor Program in Artificial Intelligence, Chang Gung University, Taoyuan 33302, Taiwan; kaiyiling3@gmail.com; 2 School of Medicine, Chang Gung University, Taoyuan 33302, Taiwan; cch1966@gmail.com (V.C.-H.C.); russell.tsai@gmail.com (Y.-H.T.); 3 Department of Psychiatry, Chang Gung Memorial Hospital, Chiayi 61363, Taiwan; 4 Department of Diagnostic Radiology, Chang Gung Memorial Hospital, Chiayi 61363, Taiwan; 5 Mood Disorder Psychopharmacology Unit, University Health Network, Department of Psychiatry, University of Toronto, Toronto, ON M5S, Canada; Roger.McIntyre@uhn.ca; 6 Institute of Medical Science, University of Toronto, Toronto, ON M5S, Canada; 7 Departments of Psychiatry and Pharmacology, University of Toronto, Toronto, ON M5S, Canada; 8 Medical Imaging Research Center, Institute for Radiological Research, Chang Gung University and Chang Gung Memorial Hospital at Linkou, Taoyuan 33302, Taiwan

**Keywords:** deep learning, chemotherapy-induced cognitive impairment (CICI), residual neural network, densely connected convolutional networks

## Abstract

Breast cancer is the most common female cancer worldwide, and breast cancer accounts for 30% of female cancers. Of all the treatment modalities, breast cancer survivors who have undergone chemotherapy might complain about cognitive impairment during and after cancer treatment. This phenomenon, chemo-brain, is used to describe the alterations in cognitive functions after receiving systemic chemotherapy. Few reports detect the chemotherapy-induced cognitive impairment (CICI) by performing functional MRI (fMRI) and a deep learning analysis. In this study, we recruited 55 postchemotherapy breast cancer survivors (C+ group) and 65 healthy controls (HC group) and extracted mean fractional amplitudes of low-frequency fluctuations (mfALFF) from resting-state fMRI as our input feature. Two state-of-the-art deep learning architectures, ResNet-50 and DenseNet-121, were transformed to 3D, embedded with squeeze and excitation (SE) blocks and then trained to differentiate cerebral alterations based on the effect of chemotherapy. An integrated gradient was applied to visualize the pattern that was recognized by our model. The average performance of SE-ResNet-50 models was an accuracy of 80%, precision of 78% and recall of 70%; on the other hand, the SE-DenseNet-121 model reached identical results with an average of 80% accuracy, 86% precision and 80% recall. The regions with the greatest contributions highlighted by the integrated gradients algorithm for differentiating chemo-brain were the frontal, temporal, parietal and occipital lobe. These regions were consistent with other studies and strongly associated with the default mode and dorsal attention networks. We constructed two volumetric state-of-the-art models and visualized the patterns that are critical for identifying chemo-brains from normal brains. We hope that these results will be helpful in clinically tracking chemo-brain in the future.

## 1. Introduction

As reported by the American Cancer Society, the three most common cancers in women are breast, lung and colorectal cancers, accounting for 50% of all new diagnoses; among all three cancers, breast cancer is the most common, accounting for 30% of female cancers [1]. Breast cancer has long been in the top five of female cancer-related deaths, and the latest survey revealed that it was the third-leading cause of cancer-related death in 2019, according to the Ministry of Healthcare, Taiwan [2]. Treatment modalities for breast cancer include surgical removal, radiotherapy, hormone therapy and chemotherapy. Benefiting from these techniques, the average 5-year relative survival rate for women with breast cancer is 90% among all races [1]; as a result of prolonged survival, the prognosis after treatments should be carefully considered.

Among all treatment modalities, previous studies reveal that breast cancer survivors who underwent chemotherapy might complain about cognitive impairment during and after cancer treatment. The word “chemo-brain”, or cognitive-induced cognitive impairment (CICI), is used to describe the alterations in cognitive function after receiving systemic chemotherapy. The impacted cognitive functions include memory, executive function, processing speed and reaction time. CICI is currently recognized as a common adverse effect in patients who were administered chemotherapeutic agents [3,4].

Magnetic resonance imaging (MRI) techniques are a safe and noninvasive method to investigate functional and structural alterations in the human brain. A specific technique called blood oxygenation level-dependent functional magnetic resonance imaging (BOLD-fMRI) has been widely applied to investigate cerebral function. Numerous studies discovered changes in cerebral function in breast cancer survivors after chemotherapy compared to healthy women using fMRI techniques. fMRI is categorized into two types: task-based fMRI and resting-state fMRI (rs-fMRI). Compared to task-based fMRI, rs-fMRI simplified the experimental design by requesting patients to relax and clear their mind during the MRI scan without following any instructions, which makes the whole experiment easier, more reproducible and friendlier to implement in clinical situations.

Contemporarily, the construction of a deep learning model for visual tasks on medical images has become prosperous. The convolutional neural network (CNN), a class of deep learning that was originally proposed by Yann LeCun in 1989 [5], imitates the connectivity pattern of the animal visual cortex and captured the attention of international researchers after it achieved great success on ImageNet classification [6,7]. The idea of CNN is similar to a distiller for information-distillation operations [8]. For each convolutional layer in the CNN, certain information is extracted from input images and passed to the next layer. As the procedure is repeated several times, the useful information is refined and amplified for a target. To date, CNNs achieved tremendous success in computer vision and pattern recognition and are capable of capturing subtle patterns from high-dimensional data that might be ignored by traditional analytical methods.

From a clinical perspective, the construction of an accurate deep learning model is insufficient, and a diagnosis should be supported with evidence suggesting that a visual explanation of a deep learning model is essential when applying a model to predict certain diseases. Many researchers developed numerous algorithms to decipher the black box of deep learning using different approaches [9]. These algorithms provide us with meaningful insights into how a deep learning system made the decision.

In this study, our goal was to establish objective 3D deep learning models that differentiate cerebral alterations based on the effect of chemotherapy and to visualize the pattern that was recognized by our model. We modified two state-of-the-art CNN models into 3D and applied a visual explanatory algorithm using the TensorFlow and Keras [10,11] higher application programming interface (API) to achieve these goals. To the best of our knowledge, this article is the first to explain the internal logic of a CNN model when distinguishing chemo-brain.

## 2. Materials and Methods

### 2.1. Participants

One hundred and twenty female participants were recruited from Chiayi Chang Gung Memorial Hospital. The participants who were diagnosed with breast cancer and received systemic chemotherapy were assigned to the C+ group (N = 55), and the remaining sex-matched individuals without a breast cancer history were assigned to the HC group. Based on the inclusion criteria, patients with breast cancer included females aged 20 to 55 years with pathologically proven primary breast cancer. The exclusion criteria for patients with breast cancer included end-stage breast cancer, comorbidities with other cancers, treatment with radiation therapy before the investigation, signs of brain metastasis or other brain insults, a previous diagnosis of neuropsychiatric disorders or substance use and an inability to undergo a MRI scan. The same exclusion criteria were used for the HC group in addition to having no evidence of breast cancer. This study was approved by the Institutional Review Board of Chang Gung Memorial Hospital, Chiayi, Taiwan. (Nos. 104-5082B, 201700256B0 and 201702027B0). All methods were carried out in accordance with relevant guidelines and regulations. Written informed consent was obtained from all participants

### 2.2. MRI Acquisition

Before the examination, all 120 subjects were instructed to relax but remain alert during the fMRI scan. fMRI acquisition was performed using a 3 T MRI scanner (Verio, Siemens, Germany) at Chiayi Chang Gung Memorial Hospital with a gradient echo planar imaging (EPI) sequence. The scanning parameters were set to a TR/TE = 2000/30 ms, flip angle = 90°, NEX = 1, FOV = 220 × 220 mm^2^, matrix size = 64 × 64 and voxel size = 3.4 × 3.4 × 4 mm^3^, and 31 axial images were acquired to cover the whole brain volume. Each rs-fMRI run contained 300 image volumes, and the total scan time was approximately 10 min.

### 2.3. Feature Engineering

In this study, we used the mean fractional amplitude of low-frequency fluctuations (mfALFF), which was extracted from functional MRI data, as our input feature. The amplitude of low-frequency fluctuations (ALFF) is physiological information that represents cerebral activity in certain areas. The mean fractional ALFF (mfALFF) is the normalized mean ALFF, and therefore, mfALFF provides a more specific measure of low-frequency oscillatory phenomena than ALFF.

All functional MRI data were preprocessed using the following procedures to acquire mfALFF: slice-timing correction, motion correction, normalization and spatial smoothing with statistical parametric mapping 12 software (SPM12; Wellcome Department of Cognitive Neurology, London, UK). For each subject, if the results of six head motion parameters surpassed 1 mm of translation or 1° of rotation, they were excluded from this study. All participants mentioned previously met the criteria and did not present significant movements. After motion correction, data were normalized to the standard Montreal Neurological Institute (MNI) space and resampled to isotropic 3 mm voxels. Finally, the data were smoothed with a 6 mm full-width half-maximum Gaussian kernel to amplify the signal-to-noise ratio. The Resting-State Data Analysis Toolkit [12] (version 1.8) was used to extract mfALFF from preprocessed fMRI data with a band pass filter of 0.01–0.12 Hz.

### 2.4. SE-Residual Neural Network and SE-DenseNet

Convolutional neural networks (CNNs) are a class of deep learning methods that are commonly implemented to visualize and analyze medical images. A recent study revealed that the performance of CNNs in visual tasks is improved by a very deep architecture [13]; however, a deeper architecture often encounters the vanishing-gradient problem when using the gradient-based method and backpropagation [14,15], which obstructs the convergence of the network. Two major state-of-the-art architectures use different mechanisms to resolve the vanishing gradient problem and thus help us construct a deeper network. A deep residual neural network (ResNet) was first proposed in 2015 and uses the idea of skip connection and identity mapping, which provide residual connections directly to earlier layers to build a deeper network. In 2016, the builders of ResNet made some minor modifications to ResNet called the full preactivation form, which results in better performance of ResNet [13,16]; DenseNet debuted in 2016 and uses the idea of dense connections and feature reuse, which concatenate the outputs from the previous layer to current convolutional outputs. Unlike ResNet that uses summation to receive information from the previous layer, the usage of concatenation forms a dense circuit of the pathway that generates better gradient flow and fewer training parameters than ResNet [17].

Squeeze and excitation block (SE-block) was integrated with our volumetric models to form SE-ResNet-50 and SE-DenseNet-121 models and to improve the performance of the ResNet and DenseNet models further [18]. SE-block is a building block for convolutional neural networks that can be embedded in existing state-of-the-art networks, according to the original paper. Following the instructions of the original paper, SE-ResNet is another modification of ResNet that integrates a SE-block with a skip connection. For DenseNet, we embedded the SE-block in the dense block, and the SE-ResNet embedded the SE-block in the skip connection.

In the present study, SE-ResNet-50 and SE-DenseNet-121 with a growth rate of 16 were constructed using the Tensoflow and Keras higher API. Our data were split into 5 C+ subjects and 5 HC subjects as our test set; the remaining data were trained and validated using 10-fold cross validation. The input size for both models was set to 64 × 64 × 64 × 1; the hyperparameter for the fitting model was predefined through the grid-search method; optimization was performed using the SGD optimizer with a learning rate of 1e-2; the batch size was set to 5. The l2 regularization factor was predefined as 1e-4, and a random rotation data method for data augmentation was implemented on the axial view before the epoch was initiated to prevent overfitting. The overall training epochs were set to 200 epochs. After training was complete, we used a well-trained model to evaluate the test set. The accuracy, precision, recall, area under the curve (AUC) and F1-score were recorded to evaluate performance.

### 2.5. Visualization through an Integrated Gradients Algorithm

We implemented an algorithm called integrated gradients for visual explanations and to understand which regions of the brain have the greatest contributions to differentiating survivors after chemotherapy and healthy control subjects. The integrated gradients method assesses which pixel (voxel in our case) in the input feature is attributed to the model prediction [19]. This algorithm can be implemented easily under TensorFlow without any modifications to the model. The original formula of integrated gradients is provided in the following equation: $IntegratedGrads_{i}\left(x\right)∷=\left(x_{i}-x\prime_{i}\right)\;\int_{\alpha =0}^{1}\frac{\partial F\left(x^{\prime }+\alpha \times \left(x-x^{\prime }\right)\right)}{\partial x_{i}}\;d\alpha$, which is interpreted as the integrated gradients along the i^th^ dimension; x and x′ represent the original input image and the baseline image, respectively, where F is the deep network being investigated. A crucial hyperparameter that shall be determined is the baseline image. Based on the recommendation from the original paper [19], a good baseline image should have a near-zero score for the desired class. In this study, we used all-zero 3D images with shapes of 64 × 64 × 64 × 1 as the baseline image.

However, the integrated gradients method provides the importance of individual voxels for a particular example rather than the importance of all voxels in the class. We computed and normalized the individual gradient images of the C+ test set across different models to map the voxels with the greatest contributions to the C+ class. The gradient images were averaged and plotted with a threshold of 0.45.

## 3. Results

### 3.1. Demographic Characteristics

In our study, we recruited 55 postchemotherapy survivors as our C+ group and 65 sex-matched healthy subjects as our HC group. The student’s t-test was performed to investigate the differences between each group. Table 1 summarizes the demographic characteristics of all participants. Both age and years of education showed statistically significant differences at *p* < 0.05

### 3.2. Model Performance

**Figure 1:**
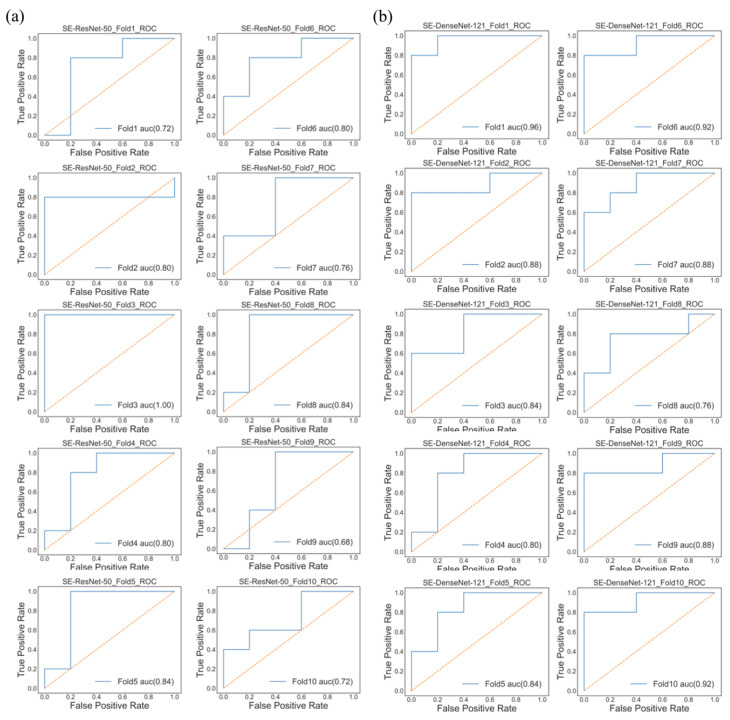
ROC curve showing the performance of each model. (**a**) The performance of the SE-ResNet-50 model had AUCs of 0.72, 0.8, 1.00, 0.8, 0.84, 0.8, 0.76, 0.84, 0.68 and 0.72. (**b**) The performance of the SE-DenseNet-121 model had AUCs of 0.96, 0.88, 0.84, 0.8, 0.84, 0.92, 0.88, 0.76, 0.88 and 0.92.

**Figure 2:**
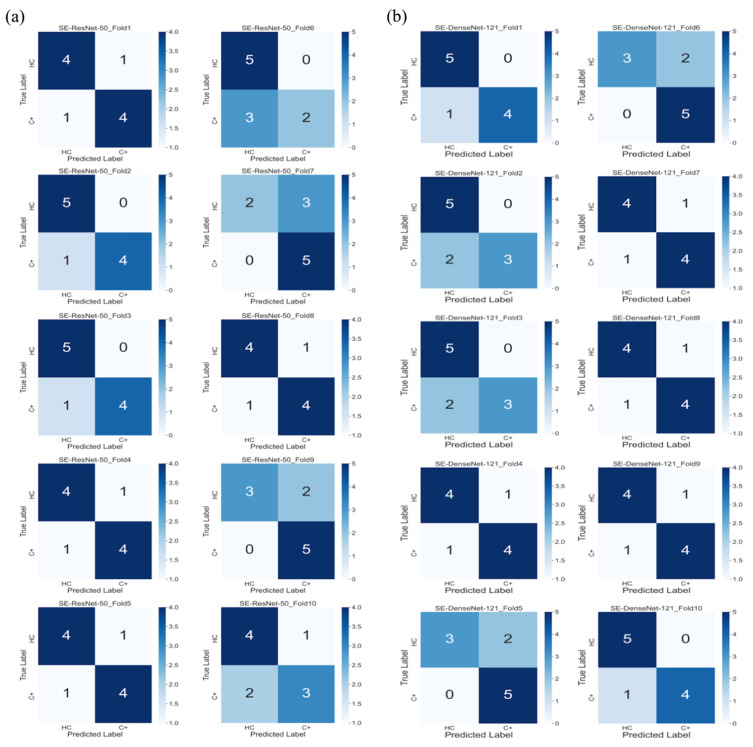
Confusion matrices of (**a**) ten SE-ResNet-50 models and (**b**) ten SE-DenseNet-121 models.

The average test results of different models are listed in Table 2. In the SE-ResNet-50 model, the average accuracy was 80% with 78% precision and 70% recall; on the other hand, the SE-DenseNet-121 model produced similar results with an average of 80% accuracy, 86% precision and 80% recall. Both of these models produced similar results at the end of the epochs, with SE-ResNet-50 achieving a slightly better performance than SE-DenseNet-121. We implemented a receiver operating characteristic curve (ROC curve) and confusion matrix for each model to understand how an individual model performed (Figure 1 and Figure 2). The mean AUCs for SE-ResNet-50 and SE-DenseNet-121 were 0.72 and 0.87 with standard deviations of 0.09 and 0.06, respectively.

### 3.3. Integrated Gradients Results

**Figure 3:**
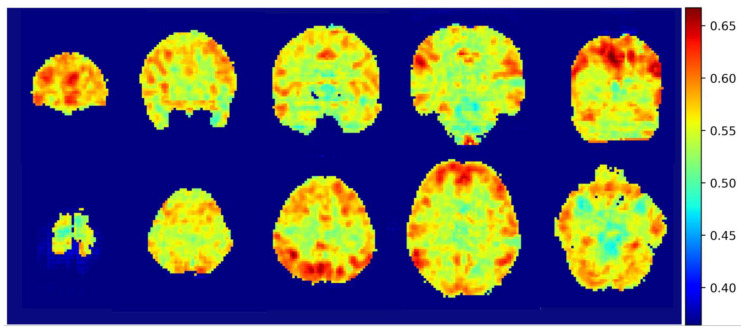
The cerebral areas that contributed to the classification and were highlighted by the integrated gradients algorithm in coronal view and axial view without thresholds.

**Figure 4:**
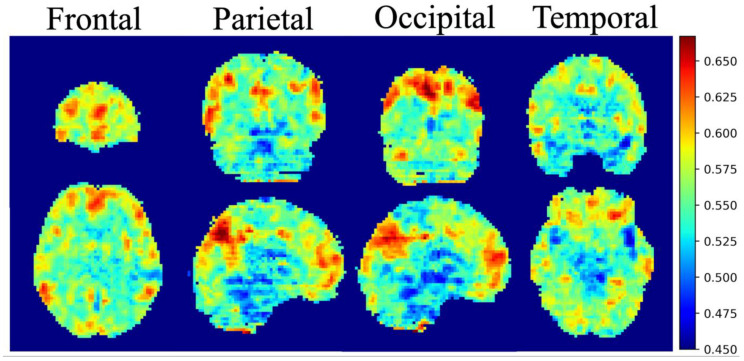
Average integrated gradients maps with a threshold of 0.45. The critical regions that are highlighted are the frontal lobe, parietal lobe, temporal lobe and occipital lobe.

The overall mean integrated gradients indicated that the predictions of postchemotherapy survivors were based on the value for the gray matter (Figure 3). The greatest contributions were observed in the frontal, temporal, parietal and occipital lobes, which represented the most crucial voxels for distinguishing postchemotherapy survivors (Figure 4).

## 4. Discussion

In this study, our deep learning model achieved a rather reliable result for our test set. We implemented the integrated gradients algorithm in 3D SE-ResNet-50 and SE-DenseNet-121 for critical feature visualization. The integrated gradients results revealed that the whole brain may contribute to the classification instead of specific cerebral regions. Although some previous studies suggested that chemotherapeutic agents might affect the whole brain, given our integrated gradients result, the evidence suggesting that the whole brain is injured by chemotherapy is insufficient in this study. By further adjusting the window level and window width, the most critical locations were identified in the frontal, temporal, parietal and occipital lobes. These findings are consistent with our previous work, and a detailed explanation of specific cerebral subregions is provided in our previous publication [20]. Each cerebral region specializes in certain functions. Based on accumulating evidence, human cognition is associated with multiple integrated regions, and our brain does not restrict its functions to a singular region but rather functions as a complex system. Currently, our brain is proposed to be wired in an economical manner, enabling the optimal efficiency of information transfer and cost [21,22,23], and the CICI may represent a brain network disruption after patients receive chemotherapy.

### 4.1. Default Mode Network & Dorsal Attention Network

In this study, an intriguing result was that the identified cerebral regions were very similar to the components of the default mode network (DMN) [24]. First introduced in 2001, the DMN is a large-scale intrinsic brain network that is spontaneously activated when an individual is awake and at rest. The subregions that were activated within the DMN included the prefrontal cortex, posterior cingulate cortex, inferior parietal lobule and lateral temporal cortex. The DMN is enriched with high-degree hub regions, indicating that the DMN regions may act as relay stations for distributing information across the brain [25,26].

The function of the DMN is generally accepted to be linked to self-relevant, internally directed information processing [27]. Accumulating evidence suggests that alterations in the DMN may be associated with impaired cognitive function [28,29,30]. Although the DMN has not been examined directly, many neuroimaging studies identified changes in DMN-related regions in breast cancer survivors undergoing chemotherapy [31]. Functional connectivity and graph theory analysis were also implemented to explore the relationship between the DMN and cognitive deficits after chemotherapy. By analyzing functional connectivity in resting-state fMRI, studies identified altered connectivity in some DMN regions and suggested that these regions were correlated with attention and memory impairments after chemotherapy [32,33,34]. Using a graph theory approach, studies showed that some DMN regions, particularly frontotemporal regions, are changed compared to healthy controls, which may explain the memory deficit occurring after chemotherapy [35]. Another approach implemented by Kesler et al. also provides even more evidence that DMN abnormalities in breast cancer survivors are associated with memory difficulties. The discovered disruptions may help explain the cognitive impairment experienced by patients after receiving chemotherapeutic drug administration [36]. Given the evidence, the DMN likely plays a critical role in CICI and can be regarded as a potential biomarker for distinguishing patients who may suffer from CICI [32].

Another discovery that is consistent with our previous finding is the alteration in the dorsal attention network (DAN) [37]. The concept of the attention network in the human brain was first introduced in 2006, and this network is categorized into dorsal and ventral systems [38]. These two networks are anatomically distinct cortical regions that control attention. The components of the DAN include the intraparietal sulcus (IPS) and frontal eye fields (FEF) of each hemisphere, and the function of the DAN is associated with focusing attention on external stimuli. Due to the characteristics of IPS and FEF, they are considered candidate regions involved in regulating spatial attention, saccade planning and visual working memory [39]. Apart from the DMN, the DAN is a task-positive network and has an antagonistic relationship with the DMN. The activation of the DAN suppresses the activation of the DMN, and the DMN and DAN appear to compete for resources within brain networks associated with cognitive control [40]. Unlike the DMN, fewer studies mentioned DAN alterations after chemotherapy. One study examined patients with breast cancer after treatment with neoadjuvant chemotherapy using arterial spin labeling techniques, and the results showed significantly increased cerebral blood flow in regions associated with the attention network [41]. Another longitudinal study examined breast cancer survivors one month and one year after the completion of chemotherapy. DAN alterations were observed one month after chemotherapy but partially recovered at one year postchemotherapy, suggesting that the change in the DAN may potentially be related to cognitive impairment after receiving chemotherapy [42].

Overall, the critical regions highlighted by the integrated gradients algorithm are consistent with our previous findings and are supported by other studies. We propose that our model is reliable and that these critical regions are trustworthy references for doctors in real clinical situations.

### 4.2. Limitations

A few underlying limitations of this study are that this model was trained on a small dataset, and a larger dataset is required to achieve better model performance; moreover, this model was used for binary classification problems. Another potential limitation of our study is that we did not consider the chemotherapeutic agent type and dosage. Although some studies indicated that the cause of CICI can predate the beginning of chemotherapy [43,44], the current weights of the model cannot be used to identify prechemotherapy patients.

## 5. Conclusions

In this study, we constructed two state-of-the-art volumetric models that were able to identify chemo-brains from normal brains. We also used an integrated gradients algorithm to visualize the pattern that was recognized by our model. The visual patterns that were shown in this study are consistent with our previous results and with other studies. We hope that these results will be helpful in clinically tracking chemo-brain in the future.

## Figures and Tables

**Table 1:** Demographic characteristics.

	C+ (N = 55)	HC (N = 65)	*p*-Value
Age (years, mean ± SD)	50.00 ± 8.09	44.71 ± 7.76	<0.001
Age range (years)	32–65	31–67	
Years of education	11.58 ± 3.8	13.56 ± 3.06	0.002
(mean ± SD)			

**Table 2:** Model performance.

Model/Performance	Accuracy	Precision	Recall	AUC	F1-Score
SE-ResNet-50	0.8 (0.07)	0.78 (0.13)	0.7 (0.18)	0.72 (0.09)	0.73 (0.1)
SE-DenseNet-121	0.8 (0.04)	0.86 (0.12)	0.8 (0.13)	0.87 (0.06)	0.81 (0.05)

## Data Availability

Due to the ethical approval and requirements of the data protection legislation, the data set will only be made available on a restricted basis according to the data sharing policies at the Chang Gung Memorial Hospital, Chiayi, Taiwan. Applications for access to anonymized data can be obtained by sending an e-mail to jcweng@mail.cgu.edu.tw.
